# Association between pesticide exposure and sleep health among a representative sample of US adults: evidence from NHANES 2009–2014

**DOI:** 10.1186/s12889-021-12014-x

**Published:** 2021-12-01

**Authors:** Astrid N. Zamora, Deborah J. Watkins, Karen E. Peterson, Erica C. Jansen

**Affiliations:** 1 Department of Nutritional Sciences, University of Michigan School of Public Health, Ann Arbor, MI USA; 2 Department of Environmental Health Sciences, University of Michigan School of Public Health, Ann Arbor, MI USA; 3 Division of Sleep Medicine, Department of Neurology, Michigan Medicine, Ann Arbor, MI USA

**Keywords:** NHANES, Urinary pesticide metabolites, Household pesticide use, Sleep duration, Trouble sleeping, Adult sleep health

## Abstract

**Background:**

Data suggest that pesticides interact with the melatonin receptor, which may influence sleep. However, the link between pesticides and sleep remains unexplored among the general adult population. This study evaluated unstratified and sex-stratified associations between urinary pesticide exposure (*N* = 4,478) and self-reported acute household pesticide exposure (*N* = 14,956), with sleep health outcomes within a nationally representative sample of US adults.

**Methods:**

Data from the National Health and Nutrition Examination Surveys (NHANES) 2009–2014 were combined for analysis of aim 1 and aim 2. Urinary pesticide metabolite concentrations served as biomarkers of pesticide exposure. Acute household pesticide exposure (if any chemical products were used in the home in the past seven days to control pests) was self-reported (yes/no). Insufficient sleep duration (< 7 h/night) and trouble sleeping (yes/no) were self-reported. Log-binomial regression models that accounted for complex survey weights and adjusted for confounders were used to compute prevalence ratios and 95% CI.

**Results:**

Log urinary 3-phenoxybenzoic acid (3-PBA) was related to a higher probability of insufficient sleep [1.09 (95% CI: 1.00, 1.20), *p* = 0.04] and trouble sleeping [1.14 (95% CI: 1.02, 1.27), *p* = 0.02] among males. Self-reported acute household pesticide exposure was associated with a higher probability of insufficient sleep duration [1.16 (95% CI: 1.02, 1.32), *p* = 0.03] and trouble sleeping [1.20 (95% CI: 1.01, 1.44), *p* = 0.04] in the unstratified sample. Sex-stratified findings showed that associations between acute household pesticide exposure and trouble sleeping only persisted  among males [1.69 (95% CI: 1.27, 2.24), *p* < .001].

**Conclusions:**

In summary, acute pesticide exposure may be detrimental to adult sleep health, particularly among US males.

**Supplementary Information:**

The online version contains supplementary material available at 10.1186/s12889-021-12014-x.

## Background

Poor sleep health among adults, including insufficient sleep duration and trouble sleeping, has been linked to adverse health outcomes. Insufficient sleep duration (typically defined as < 7 h/night) has been associated with mental health problems [1], metabolic dysfunction [2–4], risk of mortality [5], excessive daytime sleepiness [6], and cognitive dysfunction [7] among adult populations. Another component of poor sleep health is trouble sleeping, characterized by difficulty falling and staying asleep throughout the night [8]. Trouble sleeping is prevalent in conditions such as insomnia that disturb standard sleep patterns [9]. Moreover, the prevalence of poor sleep health is high among US adults, evidenced by approximately 36% of the population experiencing short sleep duration [10] and an estimated one-third with chronic insomnia [11].

Both insufficient sleep and trouble sleeping have been linked to environmental factors, such as proximity to noisy areas [12, 13], unhealthy dietary patterns, light exposure, alcohol and nicotine consumption, and stress [10]. Further, recent studies have demonstrated that environmental toxicants may affect sleep health. A group of toxicants that have been associated with poor sleep health among farmworkers are pesticides [14–18]. Previous studies using a variety of study designs, including case-control [19], longitudinal [20], and cross-sectional frameworks [16, 17, 21] have provided evidence linking pesticide exposure to rapid eye movement (REM) sleep behavior disorder [19], poor sleep quality [20] and insomnia [17]. Additionally, a recent experimental study found that pesticides ( specifically, carbamates) interact with melatonin (sleep/wake hormone) [22].

Though the link between pesticide exposure and sleep health among occupationally exposed groups has been well documented, limited knowledge exists on the relationship between acute pesticide exposure and sleep health among non-occupationally exposed general adult populations. Among non-occupational populations, the use of household pesticides is a common route of pesticide exposure. Studies have reported there are up to thirty heavily used household pesticides, including substances used to prevent, destroy, repel or mitigate any pest, such as insecticides [23]. Notably, the prevalence of household pesticide use within US households is high, with one study reporting that 80–90% of households regularly use pesticides in the home [24]. However, it is unknown whether exposure to pesticides among non-occupationally exposed adults is enough to affect the sleep of the general adult population. To address this gap in the literature, we examined associations between urinary pesticide metabolites as biomarkers of acute exposure (aim 1 analysis) and self-reported acute household pesticide exposure (aim 2 analysis) with sleep health outcomes among a nationally representative sample of US adults from the 2009–2014 National Health and Nutrition Examination Survey (NHANES).

## Methods

### Study population

We analyzed nationally representative cross-sectional data collected from the National Health Nutrition and Examination Survey (NHANES) from 2009 to 2014. NHANES is an ongoing survey that employs a complex multistage sampling design to obtain a representative sample of the US population during each collection cycle. NHANES collects information on demographic indicators and health outcomes through interviews, in-person examinations, and laboratory testing. Each year NHANES examines approximately 5,000 participants per round, with participants located in different counties across the US and a computer process randomly selecting some, all, or no household members. Complete details on NHANES study design, recruitment, procedures, and population characteristics can be accessed through the Centers for Disease Control and Prevention (https://www.cdc.gov/nchs/nhanes/index.htm). Briefly, NHANES study sampling consists of a four-stage design and oversamples some subgroups to increase precision.

The study sample for aim 1 and aim 2 analyses included NHANES participants from four consecutive cycles: 2009–2010, 2011–2012, and 2013–2014. For both analyses, we restricted the analytic sample to adults ≥ 20 years of age. In aim 1, we analyzed urinary pesticide metabolites and self-reported sleep outcomes. We excluded participants with missing data on urinary pesticide exposure, self-reported sleep outcomes, or other covariates that could serve as potential confounders. The final eligible sample for aim 1 analysis included 4,478 US adults ≥ 20 years with complete information on urinary pesticide concentration (exposure), self-reported sleep outcomes (outcome), and relevant potential confounders of interest. For aim 2 , we analyzed household pesticide use and self-reported sleep outcomes. We excluded participants with missing data on self-reported household pesticide use, self-reported sleep outcomes, or other covariates that could serve as potential confounders. The final analytical sample included 14,956 US adults ≥ 20 years of age with complete information on self-reported household pesticide use (exposure), self-reported sleep outcomes (outcome), and relevant potential confounders of interest.

### Urinary pesticide exposure assessment

From the spot urine samples provided by participants during the physical examination, one herbicide, 2,4-dichlorophenoxyacetic acid (2,4-D); one organophosphorus insecticide, para-nitrophenol (pNP); and one synthetic pyrethroid metabolite, 3-phenoxybenzoic acid (3-PBA), were measured. Regression analyses were only performed for the pesticide metabolites detected in at least 70% of the spot urine samples. Urine samples were analyzed at the Centers for Disease Control and Prevention (CDC) National Center for Environmental Health laboratory. Metabolite concentrations were measured through high-performance liquid chromatography/tandem mass spectrometry (HP-LC/MS) using validated laboratory methods that have been described elsewhere [25]. For analytes below the limit of detection (LOD), values were imputed as the limit of detection divided by the square root of 2. The LOD for 2–4-D was 0.15 μg/L, and 0.10 μg/L for both pNP and 3-PBA. Urinary creatinine concentrations were used to correct for urinary dilution. The creatinine concentrations were measured using a Beckman Synchron CX3 Clinical Analyzer or a Roche/Hitachi Modular P Chemistry Analyzer. A total of 4,478 participants had available samples for urinary pesticides and were eligible for the aim 1 analysis.

### Self-reported acute household pesticide exposure

For aim 2, the exposure of interest was acute household pesticide use, self-reported among adults who completed the self-administered NHANES questionnaire. To define exposure, participants responded either yes or no to the following question: “In the past 7 days, were any chemical products used in your home to control fleas, roaches, ants, termites, or other insects?”

### Self-reported sleep health outcomes

We abstracted participant responses to two questions about sleep habits to assess self-reported insufficient sleep duration and trouble sleeping. Average sleep duration was measured by asking participants: “How much sleep do you usually get at night on weekdays or workdays?” Based on the American Academy of Sleep Medicine (AASM) recommendations for US adults [26], we dichotomized responses into two categories: insufficient sleep duration (< 7 h/night) or sufficient sleep duration (≥ 7 h/night). Trouble sleeping was measured from the question, “Have you ever told a doctor or other health professional that you have trouble sleeping?” Responses to this question were dichotomized as either yes or no.

### Covariates

The following* a priori *covariates were included in aim 1 and aim 2 regression models: sex, age group, race/ethnicity, body-mass-index (BMI), marital status, poverty-income ratio (PIR), and smoking behavior. Urinary creatinine was also included in aim 1 regression models. For sex, males were considered the reference group. Age group was categorized as < 30 (reference), 30–39, 40–49, 50–59, and ≥ 60 years. Race/ethnicity was categorized as Hispanic, Non-Hispanic White (reference), Non-Hispanic Black, and Other. Body mass index (BMI) was categorized as underweight (< 18.5 kg/m^2^), normal weight (18.5–24 kg/m^2^ [reference]), overweight (25–29 kg/m^2^), and obese (≥ 30 kg/m^2^). Smoking behavior was categorized as either never smoker (reference), current smoker or former smoker. Marital status was classified as never married, married/living with a partner (reference), and other (i.e., separated, divorced, or widowed). Poverty income ratio (PIR), a measure of socioeconomic status, was used to represent the calculated ratio of household income to the poverty threshold after accounting for inflation and family size, with income values < 1 representing those below the poverty line [27]. Classification included PIR < 1 (any poverty) and the reference group, PIR ≥ 1 (no poverty). Finally, to account for variation in dilution in spot urinary samples, urinary creatinine was entered as a continuous variable in aim 1 regression models.

### Statistical analysis

We included complex sampling weights that accounted for unequal probabilities of selection, oversampling, and non-response in the NHANES survey, as recommended by the National Center for Health Statistics in all analyses. We used the Rao-Scott Chi-Square test for all binary and categorical variables to assess differences between baseline sociodemographic and lifestyle characteristics by self-reported sleep outcomes among participants included in aim 1 (*N* = 4,478). We then computed  summary statistics for six pesticide metabolites measured from spot urine samples. Next, we used the Rao-Scott Chi-Square test for all binary and categorical variables to assess differences between baseline sociodemographic and lifestyle characteristics by the concentration of urinary pesticides among participants included in  aim 1. Similarly, we used the Rao-Scott Chi-Square test for all binary and categorical variables to assess differences between baseline sociodemographic and lifestyle characteristics by self-reported sleep outcomes among participants included in aim 2 analysis (*N* = 14,956).

All urinary pesticide metabolite concentrations were natural log-transformed to correct for skewness and analyzed as continuous variables. We used log-binomial regression models to obtain unstratified and sex-stratified adjusted weighted prevalence ratios (PR) and 95% confidence interval (95% CI) for the likelihood of having insufficient sleep and trouble sleeping among participants with urinary pesticide data. We present findings for the fully adjusted model in which we controlled for urinary creatinine, sex, age, race/ethnicity, BMI, marital status, PIR, and smoking behavior. We then created three log-binomial regression models to determine unstratified and sex-stratified associations between insufficient sleep and trouble sleeping among participants with self-reported household pesticide exposure data. The first model was unadjusted (Crude Model I); we then adjusted for urinary creatinine sex, age, and race/ethnicity (Model II), and the fully adjusted model (Model III) was adjusted for sex, age, race/ethnicity, BMI, marital status, PIR and smoking behavior. Statistical tests were two-sided; significance was set at *p* < 0.05. All statistical analyses were conducted using SAS 9.4 (SAS Institute Inc., Cary, NC). The present study utilized publically available, de-identified data and was exempt from human subjects research.

## Results

Within this nationally representative analytic sample of 4,478 US adults, 34.7% of the sample had insufficient sleep duration based on AASM recommendations [26], with a mean (SE) sleep duration of 6.9 (0.04) h/night, while 26.4% had trouble sleeping. Moreover, among the 14,956 US adults with data on self-reported household pesticide exposure, 9.2% reported pesticide use in the household within the past seven days. Within this aim 2 sample, over one-third (36.1%) of the sample had insufficient sleep duration based on AASM recommendations [26], with a mean (SE) sleep duration of 7.0 (0.02) h/night, while 27.5% self-reported trouble sleeping.

### Aim 1: Analysis of urinary pesticide exposure

Table 1 demonstrates crude differences in sociodemographic and lifestyle characteristics among adults in the analysis of aim 1 with poor sleep health outcomes compared to adults with healthy sleep outcomes. Adults with insufficient sleep duration were more likely to be in midlife (40–49 years) and of a minority race/ethnicity. A higher prevalence of adults that self-reported insufficient sleep duration had a BMI < 18.5 kg/m^2 ^and ≥ 30 kg/m^2^, were current smokers, were separated, divorced, or widowed, completed between 9 and 12 years of education, and did not participate in moderate physical activity. Further, adults with insufficient sleep duration were more likely to have a PIR < 1.0 (any poverty) and live in food-insecure households (Table 1). We observed similar patterns between sociodemographic and lifestyle characteristics with the presence of trouble sleeping. However, adults with trouble sleeping were more likely to be female than male (31.0% vs. 24.5%; *p* < .0001), current or former smokers, and of non-minority race/ethnicity (Table 1).

**Table 1:** Baseline sociodemographic and lifestyle characteristics of the NHANES 2009–2014 study sample according to sleep outcomes (*N* = 4,478)

	N	Insufficient Sleep^a^ (%)	Trouble Sleeping (%)
Sex			
*Male*	2190	35.9	24.5
*Female*	2288	33.7	31.0
*p*		0.22	<.0001
Age group			
*< 30 years*	766	33.1	14.9
*30–39 years*	731	32.6	20.4
*40–49 years*	774	41.6	31.2
*50–59 years*	751	39.4	34.2
*≥ 60 years*	1456	29.1	29.7
*p*		<.0001	<.0001
Race/ethnicity			
*Hispanic*	1052	37.2	17.3
*NH White*	2015	31.4	29.4
*NH Black*	917	50.1	23.8
*Other*	494	38.8	18.3
*p*		<.0001	<.0001
BMI (kg/m^2^)			
*Underweight (BMI < 18.5)*	101	41.6	26.3
*Normal weight (BMI 18.5–24.9)*	1229	31.1	23.4
*Overweight (BMI 25.0–29.9)*	1446	33.0	26.5
*Obese (BMI ≥ 30)*	1702	38.5	28.5
*p*		0.01	0.13
Smoking Behavior			
*Never Smoker*	2506	32.4	21.7
*Current Smoker*	914	42.2	31.4
*Former smoker*	1058	34.1	33.4
*p*		0.0003	<.0001
Marital status			
*Never married*	875	33.4	24.1
*Married/living with partner*	2644	33.9	24.9
*Separated, divorced, widowed*	959	39.0	33.8
*p*		0.15	<.0001
Educational Attainment			
*< 9 years*	409	37.3	18.5
*9–11 years*	633	40.7	28.9
*12 years*	976	41.0	30.5
*> 12 years*	2460	31.4	25.2
*p*		<.0001	0.0017
Moderate Physical Activity			
*None*	2592	38.3	26.6
*Any*	1886	30.6	26.1
*p*		0.0006	0.78
Poverty-Income Ratio (PIR)			
*No Poverty (PIR ≥ 1.0)*	3233	33.5	26.0
*Any Poverty (PIR < 1.0)*	1245	39.8	27.9
*p*		0.0006	0.32
Household Food Insecurity (HFI)			
*None*	3064	32.6	25.4
*Any*	1414	41.6	29.4
*p*		<.0001	0.01

^a^ Indicates percentage of sample with insufficient sleep duration based on AASM recommendations for adults ≥ 20 years

ABR: *N* sample size; *BMI *body-mass index; *NH* Non-Hispanic; *PIR* poverty-income ratio; *HFI* household food insecurity

All *p* values obtained from the Rao-Scott χ2 test

Within the analytic sample of 4,478 participants from NHANES 2009–2014 with urinary pesticide metabolite measurements, three metabolites were detected in ≥70% of samples. Among the pesticide metabolites, 2,4-dichlorophenoxyacetic acid (2,4-D), 3-phenoxybenzoic acid (3-PBA), and para-Nitrophenol (pNP) were detected in 73.5, 84.5, and 91.7% of the samples, respectively (Table 2). The distribution of the other metabolites can be found in Table 2.

**Table 2:** Summary statistics for urinary pesticides metabolites measured from spot urine between 2009-2014 (*N* = 4,478)

	LOD	% > LOD	% < LOD	GM (SE) (u/mL)	Minimum	Q1	Q3	Maximum
2,4-D	0.15	73.5	26.5	0.30 (0.01)	<LOD	<LOD	0.53	97.9
4F-3-PBA	0.10	9.3	90.7	0.08 (0.00)	<LOD	<LOD	<LOD	25.5
3-PBA	0.10	84.5	15.5	0.56 (0.02)	<LOD	0.21	1.32	489.1
Oxypyrimidine	0.10	17.9	82.1	0.09 (0.00)	<LOD	<LOD	<LOD	39.2
pNP	0.10	91.7	8.3	0.54 (0.02)	<LOD	0.27	1.10	59.7
trans-DCCA	0.60	16.3	83.7	0.59 (0.01)	<LOD	<LOD	<LOD	614.8

ABR: *N* Sample size; *LOD* Limit of Detection; *GM* Geometric Mean; *SE* Standard Error; *Q1* Quartile 1; *Q3* Quartile 3; *2,4-D* 2,4-dichlorophenoxyacetic acid; *4F-3-PBA* 4-fluoro-3-phenoxybenzoic; *3-PBA* 3-phenoxybenzoic acid; *pNP para*-Nitrophenol; trans-DCCA: trans-3-(2,2-dichlorovinyl)-2,2-dimethyl-cyclopropane-1-carboxylic acid

Table 3 demonstrates geometric mean (GM) and standard error (SE) of the pesticide metabolites, 2,4-dichlorophenoxyacetic acid (2,4-D), 3-phenoxybenzoic acid (3-PBA), and para-Nitrophenol (pNP), and the crude differences in the geometric mean (SE) of the pesticide metabolites, according to sociodemographic and lifestyle characteristics. Among the sample, the GM (SE) for 2,4-D, 3-PBA and pNP were 0.30 (0.01) u/mL, 0.56 (0.02) u/mL and 0.54 (0.02) u/mL, respectively. Findings demonstrated that adults with a higher geometric mean concentration for 2,4-D were more likely to be male, older (≥ 60 years), and live in food-secure households. Further, the geometric mean concentration of 3-PBA was higher among adults that were former smokers and were separated, divorced, or widowed. No significant associations were detected between pNP and participant characteristics.

**Table 3:** Baseline sociodemographic and lifestyle characteristics of the NHANES 2009–2014 study sample according to urinary pesticide metabolites (*N* = 4,478)

	N	2,4-D GM (SE) (u/mL)	3-PBA GM (SE) (u/mL)	pNP GM (SE) (u/mL)
Overall	4478	0.30 (0.01)	0.56 (0.02)	0.54 (0.02)
Sex				
*Male*	2190	0.34 (0.01)	0.57 (0.03)	0.60 (0.02)
*Female*	2288	0.28 (0.01)	0.54 (0.02)	0.50 (0.02)
*p*		0.01	0.12	0.45
Age group				
*< 30 years*	766	0.29 (0.01)	0.56 (0.04)	0.60 (0.03)
*30–39 years*	731	0.27 (0.01)	0.54 (0.03)	0.55 (0.03)
*40–49 years*	774	0.30 (0.02)	0.55 (0.04)	0.54 (0.03)
*50–59 years*	751	0.30 (0.01)	0.57 (0.04)	0.48 (0.03)
*≥ 60 years*	1456	0.35 (0.01)	0.56 (0.03)	0.55 (0.03)
*p*		0.01	0.14	0.21
Race/ethnicity				
*Hispanic*	1052	0.26 (0.01)	0.52 (0.03)	0.57 (0.03)
*NH White*	2015	0.32 (0.01)	0.55 (0.02)	0.52 (0.02)
*NH Black*	917	0.30 (0.01)	0.66 (0.04)	0.63 (0.04)
*Other*	494	0.27 (0.01)	0.55 (0.06)	0.60 (0.03)
*p*		0.94	0.20	0.12
BMI (kg/m^2^)				
*Underweight (BMI < 18.5)*	101	0.30 (0.04)	0.62 (0.12)	0.56 (0.08)
*Normal weight (BMI 18.5–24.9)*	1229	0.28 (0.01)	0.51 (0.03)	0.53 (0.02)
*Overweight (BMI 25.0–29.9)*	1446	0.31 (0.01)	0.55 (0.03)	0.53 (0.02)
*Obese (BMI ≥ 30)*	1702	0.33 (0.01)	0.60 (0.02)	0.56 (0.02)
*p*		0.13	0.30	0.22
Smoking Behavior				
*Never Smoker*	2506	0.31 (0.01)	0.53 (0.02)	0.51 (0.02)
*Current Smoker*	914	0.25 (0.01)	0.57 (0.03)	0.60 (0.03)
*Former smoker*	1058	0.34 (0.02)	0.62 (0.04)	0.58 (0.03)
*p*		0.82	0.04	0.82
Marital status				
*Never married*	875	0.28 (0.01)	0.50 (0.03)	0.58 (0.03)
*Married/living with partner*	2644	0.32 (0.01)	0.55 (0.02)	0.53 (0.02)
*Separated, divorced, widowed*	959	0.29 (0.01)	0.65 (0.04)	0.55 (0.03)
*p*		0.70	0.003	0.55
Educational Attainment				
*< 9 years*	409	0.32 (0.02)	0.56 (0.05)	0.60 (0.04)
*9–11 years*	633	0.27 (0.01)	0.56 (0.04)	0.55 (0.02)
*12 years*	976	0.28 (0.01)	0.53 (0.03)	0.50 (0.02)
*> 12 years*	2460	0.32 (0.01)	0.56 (0.02)	0.55 (0.02)
*p*		0.86	0.49	0.52
Moderate Physical Activity				
*None*	2592	0.30 (0.01)	0.54 (0.02)	0.54 (0.02)
*Any*	1886	0.31 (0.01)	0.58 (0.03)	0.55 (0.02)
*p*		0.45	0.51	0.07
Poverty-Income Ratio (PIR)				
*No Poverty (PIR ≥ 1.0)*	3233	0.31 (0.01)	0.55 (0.02)	0.53 (0.02)
*Any Poverty (PIR < 1.0)*	1245	0.28 (0.01)	0.60 (0.0)	0.60 (0.02)
*p*		0.06	0.94	0.88
Household Food Insecurity (HFI)				
*None*	3064	0.31 (0.01)	0.54 (0.02)	0.53 (0.02)
*Any*	1414	0.28 (0.01)	0.62 (0.04)	0.58 (0.02)
*p*		0.02	0.17	0.20

ABR: *N* Sample size; *GM* Geometric Mean; *SE* Standard Error; *2,4-D* 2,4-dichlorophenoxyacetic acid; *3-PBA* 3-phenoxybenzoic acid; *pNP para*-Nitrophenol; *NH *Non-Hispanic; *BMI* body-mass index; *HFI* household food insecurity; *PIR* poverty-income ratio; All *p* values obtained from the Rao-Scott χ2 test

### Aim 2: Analysis of self-reported household pesticide exposure

Table 4 demonstrates crude differences in sociodemographic and lifestyle characteristics among adults in the analysis of aim 2, with poor sleep health outcomes compared to adults with healthy sleep outcomes. We found very similar patterns between sociodemographic and lifestyle characteristics and insufficient sleep, as reported in Table 1. However, there were some differences among those with trouble sleeping. To illustrate, adults with trouble sleeping were more likely to be female than male (31.9% vs. 22.9%; *p* < .0001), of non-minority race/ethnicity, and had a slightly higher educational attainment level (Table 4).

**Table 4:** Baseline sociodemographic and lifestyle characteristics of the NHANES 2009–2014 study sample according to sleep outcomes (*N* = 14,956)

	N	Insufficient Sleep^a^ (%)	Trouble Sleeping (%)
Sex			
*Male*	7382	37.4	22.9
*Female*	7574	34.9	31.9
*p*		0.01	<.0001
Age group			
*< 30 years*	2558	34.4	16.8
*30–39 years*	2454	38.2	22.7
*40–49 years*	2531	42.9	30.0
*50–59 years*	2420	38.2	35.3
*≥ 60 years*	1444	29.9	30.9
*p*		<.0001	<.0001
Race/ethnicity			
*Hispanic*	3510	38.6	18.0
*NH White*	6590	33.0	30.7
*NH Black*	3175	51.5	24.8
*Other*	1681	38.2	19.5
*p*		<.0001	<.0001
BMI(kg/m^2^)			
*Underweight (BMI < 18.5)*	385	38.9	25.7
*Normal weight (BMI 18.5–24.9)*	4114	32.5	24.2
*Overweight (BMI 25.0–29.9)*	1130	34.7	25.5
*Obese (BMI ≥ 30)*	1733	40.1	32.0
*p*		<.0001	<.0001
Smoking Behavior			
*Never Smoker*	8263	33.7	22.5
*Current Smoker*	3101	46.5	34.2
*Former smoker*	1088	33.4	33.6
*p*		<.0001	<.0001
Marital status			
*Never married*	2874	36.8	22.9
*Married/living with partner*	8717	34.5	25.9
*Separated, divorced, widowed*	1154	40.9	37.6
*p*		<.0001	<.0001
Educational Attainment			
*< 9 years*	1406	35.1	23.1
*9–11 years*	2169	40.5	27.0
*12 years*	3335	40.1	30.1
*> 12 years*	8046	34.1	27.0
*p*		<.0001	0.0033
Moderate Physical Activity			
*None*	8851	39.0	28.0
*Any*	6105	32.7	26.9
*p*		<.0001	0.27
Poverty-Income Ratio (PIR)			
*No Poverty (PIR ≥ 1.0)*	10740	35.0	27.3
*Any Poverty (PIR < 1.0)*	4216	40.5	28.5
*p*		<.0001	0.31
Household Food Insecurity (HFI)			
*None*	10324	33.9	26.0
*Any*	4632	43.5	32.3
*p*		<.0001	<.0001

^a^ Indicates percentage of sample with insufficient sleep duration based on AASM recommendations for adults ≥20 years

ABR: *N* sample size; *BMI *body mass index; *NH* Non-Hispanic; *PIR* poverty-income ratio; *HFI* household food insecurity

All *p* values obtained from the Rao-Scott χ2 test

### Regression findings

Table 5 reports associations between urinary concentrations of pesticides (*N* = 4,478) and acute household pesticide exposure (*N* = 14,956) with sleep outcomes among the respective analytic samples.

**Table 5:** Multivariable-adjusted log-binomial regression of associations between ln-transformed urinary pesticide metabolites and self-reported household pesticide use with sleep outcomes

	Insufficient Sleep^a^ PR [95% CI]			Trouble Sleeping PR [95% CI]		
	Overall	Males	Females	Overall	Males	Females
2,4-D^1^	0.97 (0.87, 1.07)	0.91 (0.78, 1.06)	1.04 (0.88, 1.22)	1.07 (0.91, 1.14)	0.97 (0.83, 1.14)	1.06 (0.92, 1.23)
*p*	0.52	0.21	0.64	0.77	0.69	0.41
3-PBA^1^	1.04 (0.96, 1.12)	1.09 (1.00, 1.20)	0.98 (0.89, 1.08)	1.09 (1.02, 1.17)	1.14 (1.02, 1.27)	1.06 (0.95, 1.81)
*p*	0.36	0.04	0.67	0.01	0.02	0.26
pNP^1^	0.98 (0.90, 1.07)	1.01 (0.90, 1.12)	0.96 (0.85, 1.09)	1.06 (0.97, 1.17)	1.06 (0.89, 1.25)	1.08 (0.97, 1.21)
*p*	0.70	0.91	0.51	0.16	0.52	0.51
Model 1						
*No exposure*	Reference	Reference	Reference	Reference	Reference	Reference
*Acute exposure*	1.24 (1.09, 1.40)	1.27 (1.02, 1.58)	1.21 (1.03, 1.46)	1.26 (1.07, 1.49)	1.66 (1.28, 2.16)	0.99 (0.79, 1.26)
*p*	0.002	0.03	0.04	0.007	<.001	0.96
Model 2						
*No exposure*	Reference	Reference	Reference	Reference	Reference	Reference
*Acute exposure*	1.20 (1.06, 1.36)	1.24 (0.99, 1.56)	1.16 (0.97, 1.40)	1.25 (1.05, 1.50)	1.68 (1.28, 2.21)	0.99 (0.77, 1.27)
*p*	<.001	0.05	0.09	0.01	<.001	0.91
Model 3						
*No exposure*	Reference	Reference	Reference	Reference	Reference	Reference
*Acute exposure*	1.16 (1.02, 1.32)	1.23 (0.97, 1.56)	1.09 (0.90, 1.32)	1.20 (1.01, 1.44)	1.69 (1.27, 2.24)	0.91 (0.70, 1.17)
*p*	0.03	0.08	0.36	0.04	<.001	0.44

ABR: *N* sample size; *PR* prevalence ratio; *CI* confidence interval; *4-D* 2,4-dichlorophenoxyacetic acid; *3-PBA* 3-phenoxybenzoic acid; *pNP para*-Nitrophenol; *BMI* body mass index; household food insecurity; *PIR* poverty income ratio; *P* values obtained from unadjusted (Model 1 only) and multivariable-adjusted log-binomial regression model; Model 1 unadjusted; Model 2 adjusted for sex, age, and race/ethnicity; Model 3 adjusted for sex, age, race/ethnicity, BMI, marital status, PIR, and smoking behavior; ^a^ Indicates insufficient sleep duration based on AASM recommendations for adults ≥20 years;^b^^1^Urinary concentration adjusted for urinary creatinine, sex, age, race/ethnicity, BMI, marital status, PIR and smoking behavior, except for when sex-stratified by sex

In fully adjusted multivariable log-binomial regression models, 3-PBA was associated with a 9% higher probability of trouble sleeping (1.09 [95% CI: 1.02, 1.17], *p* = 0.01) among the overall unstratified sample of adults (Table 5). However, sex-stratified adjusted findings demonstrated that the association between 3-PBA with trouble sleeping persisted among males (1.14 [95% CI: 1.02, 1.27], *p* = 0.02) only. Similarly, sex-stratified findings demonstrated that 3-PBA was associated with a higher probability of insufficient sleep duration (1.09 [95% CI: 1.00, 1.20], *p* = 0.04) among males only (Table 5). No other associations were statistically significant. Supplemental Figs. 1 and 2 provide a visual representation of differences in associations between urinary pesticide exposure and self-reported household use with respective sleep outcomes after sex stratification.

Unadjusted log-binomial regression models showed that adults whom self-reported acute household pesticide exposure had a 24% higher probability of insufficient sleep duration (1.24 [95% CI: 1.09, 1.40], *p* = 0.002) compared to those that did not have acute exposure. Moreover, the probability of trouble sleeping was 26% higher (1.26 [95% CI: 1.07, 1.49], *p* = 0.007) among adults that reported acute exposure versus those that did not. Our findings were slightly attenuated after adjustment for age, race/ethnicity, body-mass-index (BMI), marital status, poverty-income ratio (PIR), and smoking behavior, with those with acute household exposure having a 16% (1.16 [95% CI: 1.02, 1.32], *p* = 0.03) higher probability of insufficient sleep duration. After full adjustment, acute household pesticide exposure was associated with a 20% higher (1.20 [95% CI: 1.01, 1.44], *p* = 0.04) probability of trouble sleeping among the overall unstratified adult sample. When we stratified by sex, models adjusted for age, race/ethnicity, BMI, marital status, poverty-income ratio (PIR), and smoking behavior demonstrated that the association between acute household pesticide exposure and insufficient sleep duration differed between males and females, with males having a higher probability (1.23 [95% CI: 0.97, 1.56], *p* = 0.08) than female counterparts (1.09 [95% CI: 0.90, 1.32], *p* = 0.36), though sex-stratified findings were not statistically significant. However, associations between acute household pesticide exposure and trouble sleeping appeared to be much stronger in males than females. Males with acute exposure had a 69% higher probability of trouble sleeping (1.69 [95% CI: 1.27, 2.24], *p* < .001), while there was no association among females (0.91 [95% CI: 0.70, 1.17], *p* = 0.44) (Table 5).

## Discussion

Within the present nationally representative sample of US adults, findings demonstrated that urinary 3-PBA concentration was related to a higher probability of trouble sleeping among adults with laboratory-assessed pesticide levels. When we stratified by sex, we found that urinary 3-PBA concentration was related to a significantly higher probability of insufficient sleep and trouble sleeping among males only. In alignment, in the overall unstratified sample of US adults with self-reported data, acute household pesticide exposure was associated with a higher probability of insufficient sleep duration and trouble sleeping, and sex-stratification demonstrated that the association between self-reported acute household pesticide exposure and trouble sleeping persisted only among males.

Overall, the present study provided evidence showing that urinary 3-PBA was associated with a higher probability of insufficient sleep duration in males only. In alignment, aim 2 analysis demonstrated that the adjusted probability of insufficient sleep duration was 16% higher among the unstratified sample of adults with acute household pesticide exposure than those without acute exposure to household pesticides. To our knowledge, no other study has reported on the association between sleep duration and acute household pesticide exposure among the general population. Existing human studies on pesticide use and sleep health measures have studied groups heavily exposed to pesticides, either through occupation or through poisoning. One cross-sectional study among adults in rural Brazil found associations between self-reported pesticide toxicity (i.e., having experienced pesticide poisoning at least once in their lifetime) and overall sleep problems [28]. However, it was not determined when the pesticide poisoning occurred or whether they were recently exposed to pesticides.

There is also existing evidence linking pesticide exposure to poor sleep health among occupationally exposed groups. To illustrate, a study of 180 farmworkers from Bangladesh found that over 50% of the sample self-reported sleep troubles and insomnia [18]. Another study showed that exposure to low concentrations of organophosphate pesticides was associated with insomnia and disturbed sleep in commercial pesticide sprayers and farmers recently exposed to organophosphate agents [29]. In the present study, we similarly found that pesticide exposure was associated with trouble sleeping. However, our study represents an important extension because findings demonstrate that even acute (i.e., short-term) exposure via household use may be enough to disturb sleep. Further, we found that the associations with trouble sleeping- both urinary pesticides and self-reported - were evident only among male adults. Among males, each one unit of log-transformed 3-PBA higher was associated with a 14% higher probability of trouble sleeping, and self-reported acute household pesticide exposure was associated with a 69% higher probability of trouble sleeping. Interestingly, many of the previous studies within occupational groups were conducted only among males. For example, a study of male Egyptian pesticide applicators and formulators demonstrated that the frequency of insomnia (i.e., trouble sleeping) was high among males exposed to pesticides and found that the percentage of insomnia increased with longer exposure duration [14].

Potential mechanisms for the associations may be related to melatonin, a chronobiotic hormone that adjusts the central biological clock’s timing [30]. Experimental research has demonstrated that pesticides such as carbofuran and carbaryl have a high structural similarity to melatonin and may interfere with the melatonin pathways [22], including the sleep/wake cycle. Another study that used animal models demonstrated that exposure to carbaryl induced changes in the pineal gland function and altered serum melatonin levels [31]. However, human data on the relationship between pesticide exposure and endogenous serum melatonin levels are lacking. Data to explain the sex differences are also lacking. However, sex steroids have been shown to differentially impact the sleep health of males versus females [32]. Thus, differential study findings may be due to differences in the interactive effects between sex-steroids and pesticide metabolites in relation to sleep. To illustrate, 3-PBA levels were linked to decreases in estradiol and increases in luteinizing hormone. Thus the male endocrine function may be susceptible to pyrethroid metabolites [33]. Other studies have provided biological evidence demonstrating the impact 3-PBA can have on estrogenic activity [34–36].

This study’s strengths include a large sample size obtained from nationally representative data that incorporated four data cycles. Further, we could account for several important potential confounders. Results from analysis of urinary pesticide exposure and  self-reported household pesticide use may work in unison to provide evidence of the association between pesticides and poor sleep health, albeit there are likely other potential pesticide exposure sources other than just recent household pesticide use. However, findings must be interpreted considering several limitations. The cross-sectional design means the findings could be subject to reverse causation. Second, we did not have information on other toxicants, meaning we cannot rule out residual confounding potential by other exposures known to influence sleep health. NHANES did not collect detailed self-reported information on household pesticide use, such as types and brands of chemicals used, timing, duration, frequency, or intensity of use, which may have toxicant-specific effects on sleep outcomes. Third, study findings may be subject to confounding by season and geographic location. In particular, because our analysis did not account for season or geographic location in which participants were questioned or sampled, we could not account for seasonal and geographic variation, which may have impacted study findings. Finally, although biomonitoring has several strengths, multiple urine samples are required to accurately classify long-term exposure to chemicals with short half-lives, such as those in the present study [37]. However, the subjective exposure question used in this study captured acute exposure to household pesticides. Thus, the utility of the urine sample would align with the timing of urinary exposure.

## Conclusions

In conclusion, findings showed a high prevalence of insufficient sleep duration and trouble sleeping within this nationally representativesample of US adults. One urinary pesticide biomarker (3-PBA) was associated with insufficient sleep duration and trouble sleeping among male adults. Moreover, self-reported acute exposure to household pesticides was associated with a higher probability of insufficient sleep and trouble sleeping among all adults. After stratification by sex, findings for trouble sleeping also only persisted among males. Future research should determine underlying mechanisms explaining associations between pesticide exposure and adverse sleep health outcomes among the general population and stratified by sex. Understanding these mechanisms is imperative to  informing future interventions and policies that aim to mitigate household pesticide exposure . In addition, future research among nationally representative samples of adults should consider testing for differences in associations across sociodemographic and lifestyle characteristics, such as race/ethnicity, poverty-income ratio, and smoking behavior.

## Supplementary Information

**Additional file 1: Supplemental Fig. 1.** Forest plot of sex-stratified associations between household pesticide use and ln-transformed urinary pesticide metabolites with insufficient sleep duration. **Supplemental Fig. 2.** Forest plot of sex-stratified associations between household pesticide use and ln-transformed urinary pesticide metabolites with trouble sleeping.

## Data Availability

This study utilized publicly available data from the National Health and Nutrition Examination Survey. This data can be accessed using the following link: https://www.cdc.gov/nchs/nhanes/index.htm

## Abbreviations

N: Sample size
NH: Non-Hispanic
PIR: household poverty-income ratio
HFI: household food insecurity
AASM: American Academy of Sleep Medicine
LOD: Limit of Detection
GM: Geometric Mean
SE: Standard Error
Q1: Quartile 1
Q3: Quartile 3
2,4-D: 2,4-dichlorophenoxyacetic acid
4F-3-PBA: 4-fluoro-3-phenoxybenzoic
3-PBA: 3-phenoxybenzoic acid
pNP: para-Nitrophenol
trans-DCCA: trans-3-(2,2-dichlorovinyl)-2,2-dimethyl-cyclopropane-1-carboxylic acid
