# Supplementary material for: Association between pesticide exposure and sleep health among a representative sample of US adults: evidence from NHANES 2009–2014
Source: BMC Public Health. 2021 Dec 1;21:2199. doi: 10.1186/s12889-021-12014-x (PMC8638511; doi:10.1186/s12889-021-12014-x)
Supplement: Supplementary file 1 — Additional file 1: Supplemental Fig. 1. Forest plot of sex-stratified associations between household pesticide use and ln-transformed urinary pesticide metabolites with insufficient sleep duration. Supplemental Fig. 2. Forest plot of sex-stratified associations between household pesticide use and ln-transformed urinary pesticide metabolites with trouble sleeping. [file 12889_2021_12014_MOESM1_ESM.docx]

**Supplemental Figure 1.** Forest plot of sex-stratified associations between household pesticide use and ln-transformed urinary pesticide metabolites with insufficient sleep duration


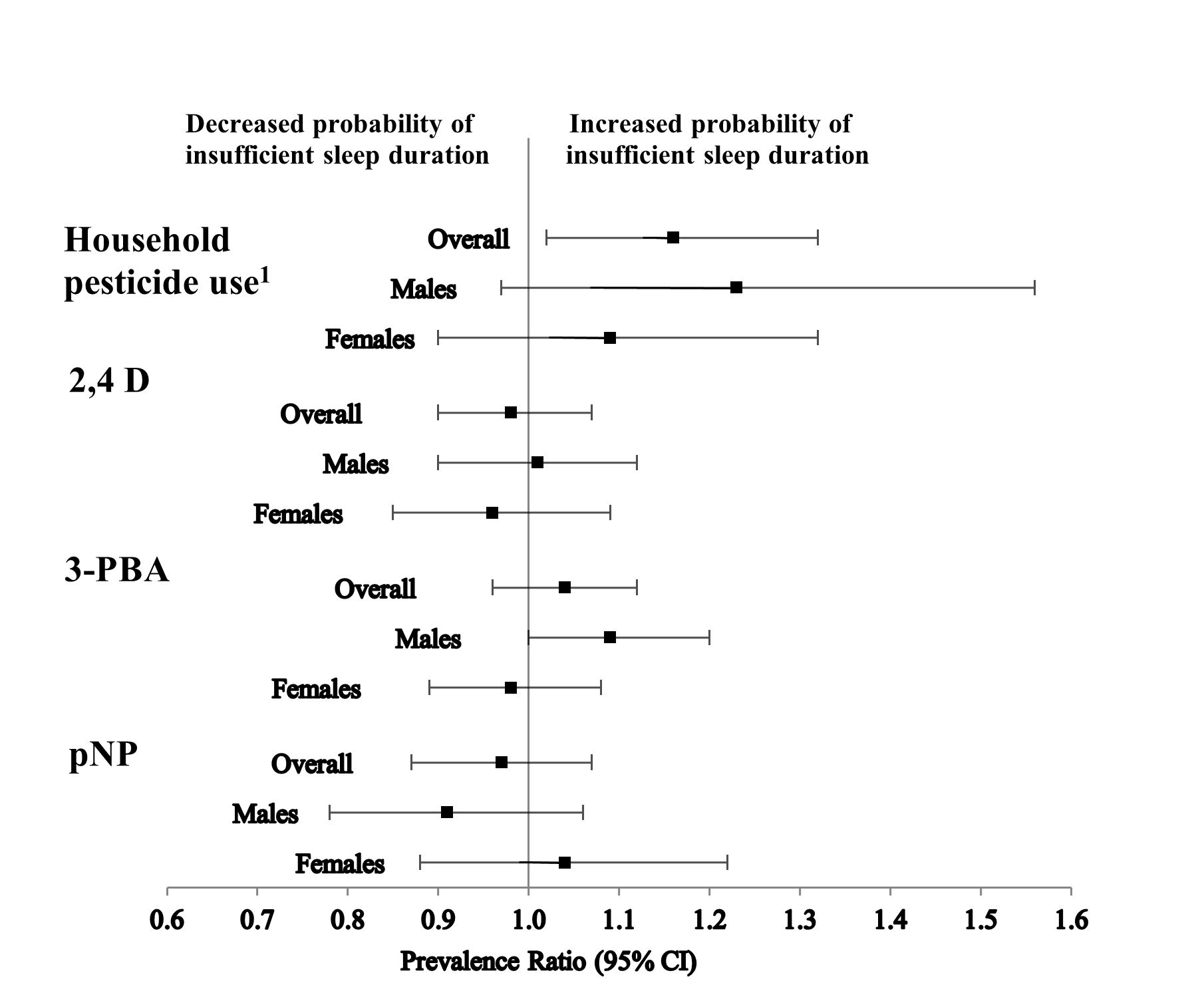


^1^Estimates obtained from model three; ABR: PR: prevalence ratio; CI: confidence interval;

4-D: 2,4-dichlorophenoxyacetic acid; 3-PBA: 3-phenoxybenzoic acid; pNP: *para*-Nitrophenol

**Supplemental Figure 2.** Forest plot of sex-stratified associations between household pesticide use and ln-transformed urinary pesticide metabolites with trouble sleeping


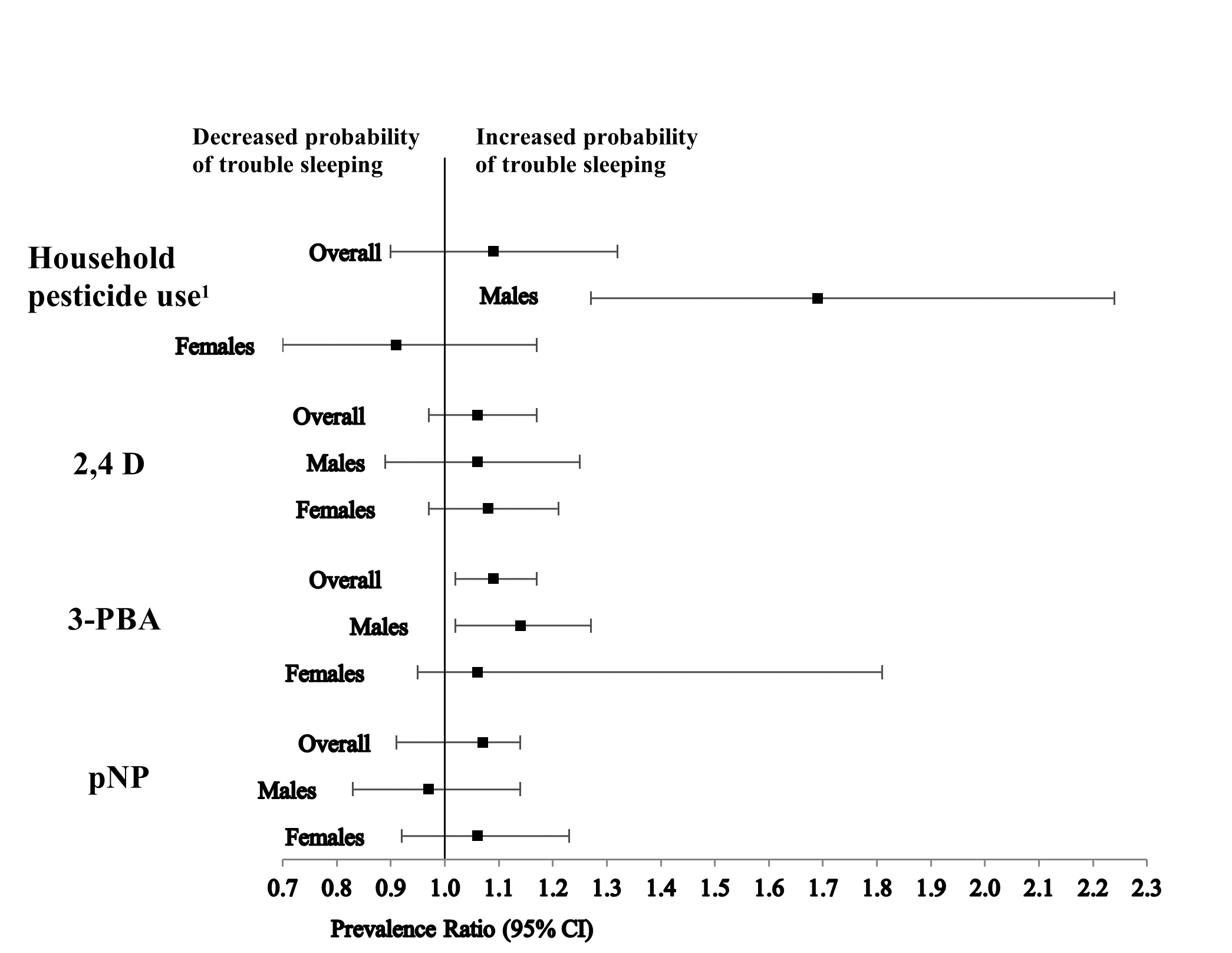


^1^Estimates obtained from model three; ABR: PR: prevalence ratio; CI: confidence interval;

4-D: 2,4-dichlorophenoxyacetic acid; 3-PBA: 3-phenoxybenzoic acid; pNP: *para*-Nitrophenol
